# Deflection of mini implants from its intended path of placement on varying bone densities

**DOI:** 10.4317/jced.59903

**Published:** 2022-12-01

**Authors:** Indra Annamalai, Khaniya Bharathan, Prema Anbarasu, Saravana-Kumar Subramanian

**Affiliations:** 1 Department of Orthodontics, Chettinad Dental College and Research Institute, Tamil Nadu, India; 2 Department of Orthodontics, RVS Dental College and Hospital, Tamil Nadu, India

## Abstract

**Background:**

Knowledge of bone density in maxilla and mandible will allow the clinician to plan the anchorage strategies and placement of implants with necessary precautions. The study aims to evaluate the deflection changes of titanium alloy self-drilling mini implants from the intended path that occurs during placement in varying bone densities.

**Material and Methods:**

63 titanium alloy self-drilling mini implants of the lengths 6mm, 8mm, and 10mm with diameter of 1.3mm were placed in three homogenous solid rigid polyurethane foam (saw bone) with bone densities of 20pcf, 30pcf, and 40pcf simulating anatomic sites in maxilla and mandible. 7mini implants of each length in all bone densities were tested for study. The implants were inserted perpendicularly into artificial bone block held in a custom made stand. The bone blocks were then radiographically exposed and the deviation of the long axis of the implantfrom a true vertical line was measured.

**Results:**

There was a decrease in deflection of the mini implant with increase in density. On the other hand, increase in length resulted in increase in the amount of deflection.

**Conclusions:**

Longer mini implants can be used in less dense bone as in maxilla, whereas shorter mini implants can be used in high dense bone as in mandible to increase the stability and success rate of implants. Bone density and implant length play a role in deflection of mini implant from its intended path of insertion.

** Key words:**Orthodontic Mini implants, deflection, bone density, anchorage.

## Introduction

Orthodontic treatment involves the application of optimal force systems to teeth, with the intention of inducing a biological response that results in tooth movement (1). However, even a small reactive force can cause undesirable movements, hence it is important to have absolute anchorage to avoid them. Miniscrew implants (MSIs) are a treatment adjunct designed to provide absolute skeletal anchorage in orthodontics. They have gained in popularity due to their simplicity in placement, low cost, patient-acceptance and ability to eliminate patient compliance issues in treatment (2). The quality of bone plays a major role when deciding on mini-implant placement site as it becomes most important factors for achieving good primary stability (3). Therefore it is important for a clinician to understand the bone density and varying cortical bone thickness throughout the maxilla and mandible. Anterior regions of the maxilla contain significantly higher proportions of cortical bone than the posterior maxilla, while the reverse is true in the mandible (4,5). As a general guideline, cortical bone thicknesses reach approximately 1.0-2.2mm in the anterior alveolar process of the maxilla and hard palate. The cortical bone becomes significantly thinner in the posterior maxilla and tuberosity region, often reaching thicknesses of less than 1mm. Cortical bone thickness is on average 1.0-1.5mm in the anterior interradicular sites of the mandible, increases to 1.5-2.5mm in the canine and premolar interradicular areas, and can reach thicknesses greater than 3.0mm in the mandibular molar and retromolar region (6).

Whenever mini implants are inserted into bone, due to the resistance offered by the bone of varying density and cortical bone thickness, the implants are liable to undergo deviation from its original path of insertion. This deflection or deviation from the bone is dependent on both the dimensions of the implant and bone density which ultimately can lead to fracture or failure of the mini implant.

According to Kuroda *et al*. (7), root proximity is one of the major risk factors for failure of mini implants. Placement of a mini screw too close to a root can also result in insufficient bone remodelling around the screw and transmission of occlusal forces through the teeth to the screws leading to implant failure. Since majority of the mini implants for orthodontic usage are placed in inter-dental areas, a slight deflection from the intended path can thus affect their success. Therefore this study attempts to radiographically evaluate the deflection of titanium alloy self-drilling mini implants from the intended path during its placement as well as to evaluate the role of bone densities and implant lengths on deflection.

## Material and Methods

The study was reviewed and approved by the Institutional Review Board (approval number 141/IHEC/Jan. The sample size of 63 was decided for the study using power analysis by GPower3.0.5 software. Sixty three Absoanchor self-drilling, mini implants made of Titanium-6Aluminium-4Vanadium (Ti-6Al-4V) alloy implants from Dentos® Korea,of the following dimensions were used for the experiment. Titanium mini implant- Length 6mm, 8mm and 10mm with diameter 1.3mm– 21 no’s each were used for the study. Three homogenous solid rigid polyurethane foam (saw bone) with different bone density such as 20 pcf, 30 pcf, 40 pcf- 21 no’s each, were used in this study which simulate anatomic sites for clinical insertion of mini implants in maxilla and mandible. Bone blocks were segregated forimplant insertion such that one block had one mini screw. Saw bones have the biological properties similar to those of natural bone. Artificial bone, which is composed of synthetic, homogeneous materials, has been shown to be a good substitute for jaw bone (8).

**Figure 1:**
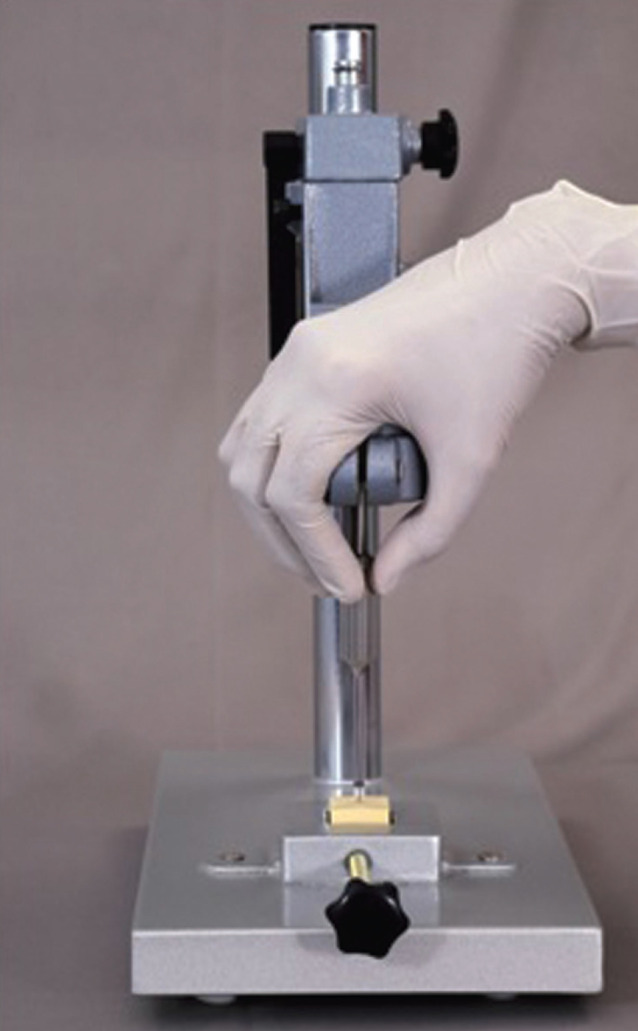
Custom made stand for placement of mini implants.

A long handle implant driver is used for insertion. The implant, implant driver and the bone block were held perpendicular to each other in the custom made stand (Fig. 1), made in such a way to enable adjustment of the bone block and driver in vertical plane. In order to confirm the point of insertion of the implant was truly horizontal, a spirit level was placed on the surface of the block before insertion. The mini implant was inserted into the bone block by slow continuous manual insertion. Similarly, all the remaining implants were also inserted one mini implant per bone block. Once the mini implants were inserted, a digital radiograph was taken for each of the blocks individually. A G.E Discovery XR656 digital radiographic machine with the X-ray source 100cm from the object set at 80kV and 292mAs was used with radiographic exposure time of 1milli second. The bone blocks were placed at the centre of the X-ray beam path. A spirit level was used to ensure that the blocks were not inclined.

**Figure 2:**
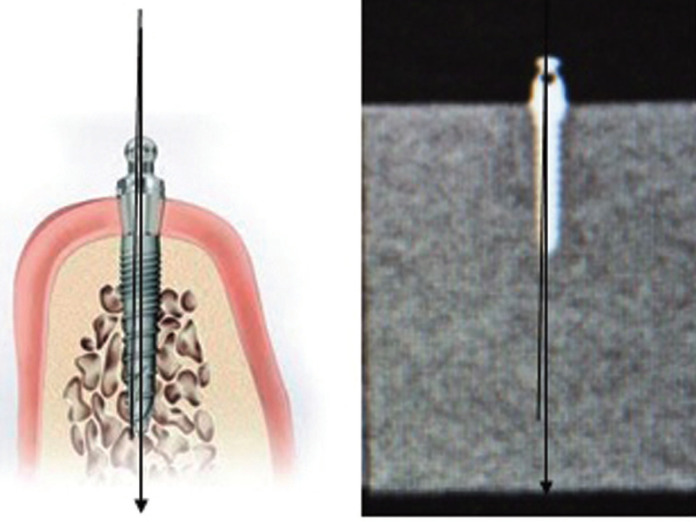
a) Pictorial representation of deflection of mini implants. b) Radiographical evaluation of mini implants.

The radiographic image obtained was adjusted for optimum contrast and magnification prior to obtaining the mini implant deflection values. Image analysis was done using the G.E. Media Viewer software as the tool for measuring the implant deflection (Fig. 2). The long axis of the mini implant was considered as a line joining the apex and the tip of the implant. A true vertical line passing through the centre of point of insertion of the mini implant was used to obtain the degree of deviation of its long axis upon insertion into the bone. The procedure was thus repeated for all the mini implants.

-Statistical analysis

Descriptive statistics, including the mean value and standard deviation of the deflection value for different implant lengths and bone densities were calculated. For significant differences, the data were evaluated using a one-way analysis of variance (ANOVA) test, followed by the post hoc test. SPSS 17.0 was used to find estimates and significance. The mean difference is significant at 0.05 level. Correlating the implant lengths and bone densities, maximum and minimum deflection was determined using Response Surface Method analysis.

## Results

The descriptive statistics of observed deflection showing the mean values of deflection of the implants with varying bone densities and varying implant length with their respective standard deviation (Table 1). All mini implants underwent deflection upon insertion with a maximum mean deflection of 1.1 degrees and a minimum of 0.6 degrees. ‘A test of between subjects’ effects was done to assess the influence of length and density and also the combined effects of length and density on deflection. The influence of length and density was found to be statistically significant. The influence of combined effects of length and density was found to be non significant.

**Table 1:**
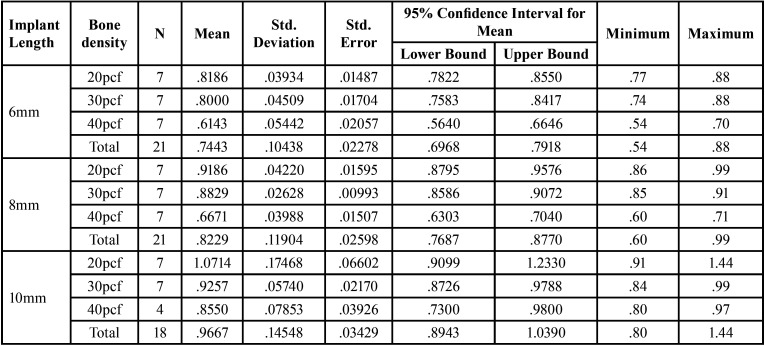
Descriptive Statistics: Effect of Bone Density and Implant Length on Deflection.

**Figure 3:**
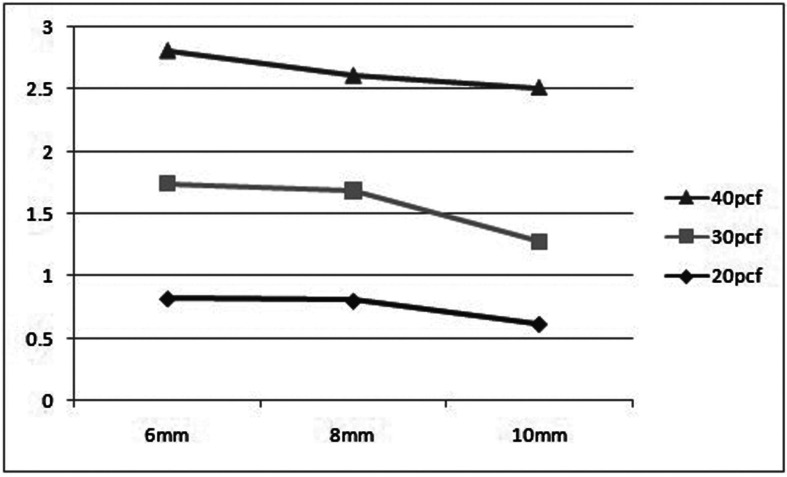
Graphical representation of deflection of mini implants with varying bone densities and length.

For significant differences, the data were evaluated using a one-way analysis of variance (ANOVA) test ( Table 2, Table 3). After evaluating an overall statistically significant difference in group means using one way – ANOVA, Post Hoc Tests are carried out to determine the difference between groups. There is a constant decrease in deflection with increase in density. 20pcf showed maximum deflection followed by 30pcf and the least was seen in 40 pcf . Similar results were obtained for implants of all dimensions (Table 4). There is a constant increase in deflection with increase in length. 10mm mini implant showed maximum deflection followed by 8mm and the least was seen in 6mm. Similar results were obtained in all the bone densities (Table 5). Deflection of mini implant with different dimension (6mm X 1.3mm, 8mmX 1.3mm, 10mmX 1.3mm ) in different bone densities (20pcf, 30pcf, 40pcf) (Fig. 3).

**Table 2:**
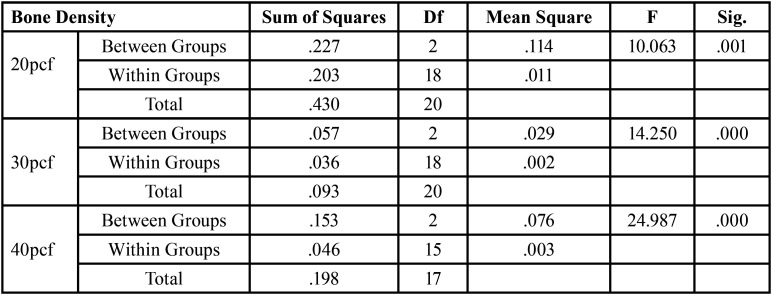
ANOVA Test for Varying Bone Densities.

**Table 3:**
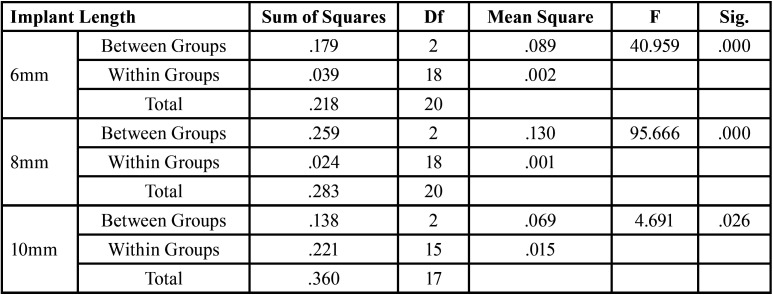
ANOVA Test for Varying Implant Lengths.

**Table 4:**
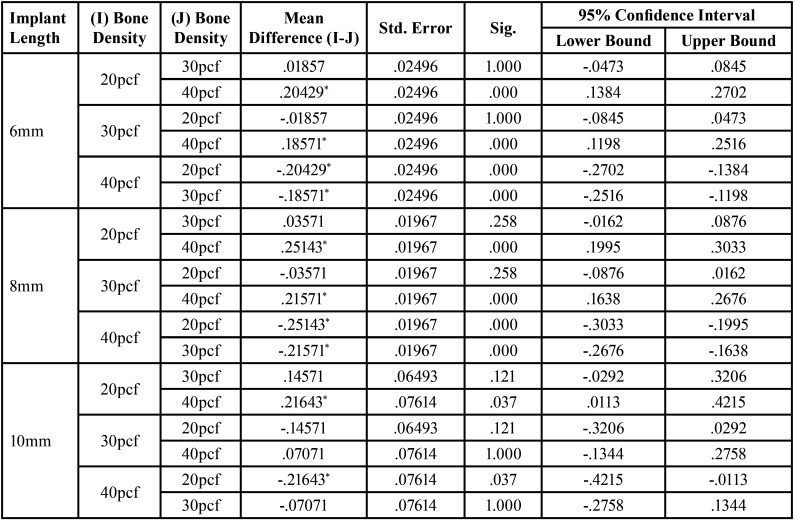
Post Hoc Tests for Deflection of Mini Implant With Varying Bone Density.

**Table 5:**
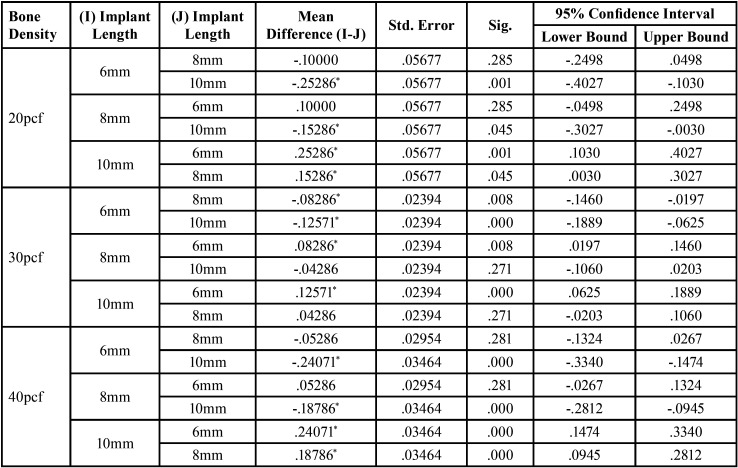
Post Hoc Tests for Deflection of Mini Implant With Varying Implant Length.

The mean deflection of a mini implant that can occur in each bone density irrespective of length of the mini implant are as follows: Minimum deflection of 0.8˚ and maximum of 1.0˚ was seen in 20pcf. Minimum deflection of 0.7˚ and maximum of 0.9˚ was seen in 30pcf. Minimum deflection of 0.6˚ and maximum of 0.8˚ was seen in 40pcf 

The mean deflection of mini implants of varying lengths irrespective of the bone density it is inserted are as follows: 6mm mini implant deflected to a maximum of 0.8˚ and minimum of 0.6˚. 8mm mini implant deflected to a maximum of 0.9˚ and minimum of 0.7˚. 10mm mini implant deflected to a maximum of 1.0˚ and minimum of 0.9˚

**Figure 4:**
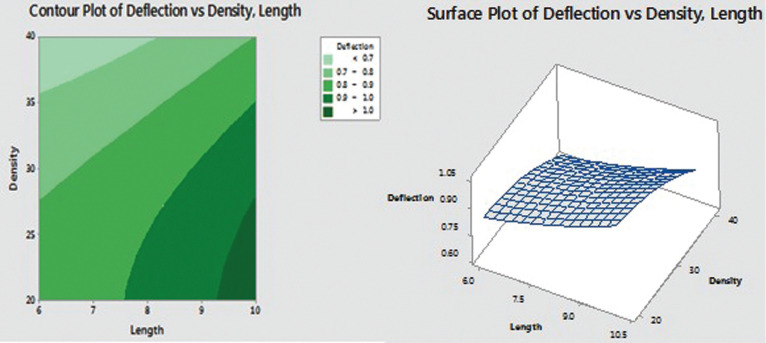
Response Surface Method analysis.

Correlating the lengths and densities maximum and minimum deflection was determined using Response Surface Method analysis (Fig. 4). Response Surface Method analysis provided the following quadratic equation to find optimum solution.

The following graphs are generated for the optimization:

Deflection = 0.593 - 0.0208 Length + 0.0214 Density + 0.00522 Length*Length

- 0.000491 Density*Density - 0.000434 Length*Density

Correlating the lengths and densities the maximum deflection was seen in 10mm mini implant in 20pcf was about 1.05˚. Correlating the lengths and densities the minimum deflection was seen in 6mm mini implant in 40pcf was about 0.6˚.

## Discussion

Temporary anchorage devices have added a whole new dimension to orthodontic treatment, allowing tooth movements to be carried out which were earlier thought difficult or impossible (9). Most commonly mini screws are made of stainless steel and commercially pure titanium and its alloys. Titanium screws have the advantage over the stainless steel as they have high bioactivity and more flexibility that improve integration and mechanical fixation.

Roberts *et al*. (10), indicated that titanium implants provided firm osseous anchorage for orthodontics. Hence Grade 5 titanium (Ti-6Al-4V) implant material was chosen for the present study.

Mini implants are available in different lengths (5 - 12mm) and diameters (1.2 – 2mm) to accommodate placement at different sites in both jaws. Deguchi *et al*. (11), recommended that mini screws less than 1.5mm in diameter could reduce the failure rate in cases where the roots of the adjacent teeth are too close. Poggio *et al*. (12), in his study showed that 1.2 – 1.3 mm diameter mini implants were placed safely when less than 3.5mm of interradicular space is available. Thinner implants lead to risks of fracture while thicker implants makes root contact more probable (13). Hence in this study commonly used dimensions of implants have been used for evaluation and comparison of deflection.

Previous studies had shown differences in the bone densities of the 4 anatomical regions in the mouth were significant, with the anterior mandible yielding a higher mean bone density value, followed by the anterior maxilla, the posterior mandible, and the posterior maxilla (14). Detailed information on bone density will help us to identify suitable implant sites, thereby improving the success rate of the procedure. In this study artificial bone block (Sawbones; Pacific Research Laboratories Inc, Wash) were used. In numerous previous studies (15), wood, polyvinyl chloride, and porcine bone were used as the test materials in in-vitro tests. In the present study, the artificial bone, the biological properties of which are similar to those of natural bone, is more suitable to determine the deflection of micro-implants.

Studies have shown that the placement angle of the screw can have an effect on its anchor value and the stress transmitted. Woodall *et al*. (16), through their finite element analysis and parallel cadaver study clearly demonstrated that compared to 30° and 60°, a 90° insertion angle to the bone surface showed the maximum anchorage advantage. Jasmine *et al*. (17), through their finite element analysis study showed that perpendicular insertion of mini implant in bone reduces the stress concentration and offers more stability to orthodontic loading. Hence the insertion angle was chosen as 90° for the present study.

All mini implants had deflected to varying degrees upon insertion into the bone irrespective of its length and density chosen. Correlating the lengths and densities the maximum deflection was seen in 10mm implant in 20pcf artificial bone and the minimum deflection was seen in 6mm implant in 40pcf artificial bone. By keeping length and diameter constant there was progressive decrease in deflection with increase in density of the bone (20pcf, 30pcf, 40pcf). This decreasing tendency of deflections is consistent for all the lengths of the mini implants (6mm, 8mm, 10mm).

In our study maximum deflection was seen in 20pcf rather than 40pcf artificial bone. This outcome might be explained as higher the density of bone greater the initial stability of the implant. In an *in vitro* study Abhishek Meher *et al*. (18), described similar outcomes of deflections. Greater stress and deflection was observed with 1.5mm rather than 2mm cortical bone thickness.

Furthermore , by keeping the density of the bone and diameter of implant constant, there was progressive increase in deflection of the implant with increasing length (6mm, 8mm, 10mm). This increasing tendency of deflection as length of mini implant increases is consistent for all the bone densities (20pcf, 30pcf, 40pcf). Corina *et al*. (19), in his study with prosthetic implants showed that longer implants deviated during placement. Similar outcome was seen in Jan D’haese *et al*. (20), study that shorter implants showed lesser deviation compared with longer implants which is explained by the fact that drilling deeper into the bone with a similar angle of insertion results in a higher apical deviation for a longer implant. The difference in mechanical properties between cortical bone and titanium alloy is a factor responsible for deflection of the mini implant which was exhibited in this study.

In our study also the deflection was observed at the point of entry of the mini implant into bone. Singh *et al*. (21), in their finite element study observed deformation of titanium alloy screws but not that of stainless steel screws under similar loading conditions and also that the stress pattern was greatest at the neck of mini implant in both screws. Our study is concurrent with Liu *et al*. (22), also who stated that the point of entry of the implant into the cortical bone acts as a pivot for its bending.

Longer mini implants when placed in high density bone, insertion torque increases there by chances of fracture or breakage of implant is more. Tehemar *et al*. (23), stated that predrilling to reduce the insertion torque will lead to heat generation that result in bone necrosis. Longer mini implants in high density bone will increase the failure rate by increasing the deflection of the implant as exhibited in this study.

Longer mini implants in low density bone showed maximum deflection. In order to increase the surface area and reduce the stress in the bone, length or width of the implant is increased. Tadas *et al*. (24), performed a 3- dimensional finite element analysis to evaluate the influence of implant length as well as that of bone quality, on the stress/strain in bone and implant. The results of this study suggest that bone of higher rather than lower density might ensure a better biomechanical environment for implants. Moreover, longer screw-type implants could be a better choice in a jaw with bone of low density.

In the present study three mini implants were fractured at the neck of the implant during insertion in the 40pcf artificial bone which may be due to increased torsional stress during placement leading to implant bending and fracture.

It is thought that the placement torque of self-drilling mini-implants can easily become excessive in the thick, mandibular cortical bone, which can cause the mini implant to fracture. When mini implants of different diameters produced by the same manufacturer were compared by *Pi*thon *et al*. (25), it was found that their torsional strength values increased as their diameters also increased. This means that insertion torques for installing small diameter mini-implants into high density bones is near the fracture torque, thus requiring more careful attention. Excessive torque also increases microdamage to cortical bone leading to cracks in the cortical bone immediately adjacent to the implant surface.

Understanding the biologic and mechanical aspects of mini implants in orthodontics is an essential prerequisite. Bone density and soft tissue health directly affect implant stability. Longer mini implants can be used in less dense bone as in maxilla, whereas shorter mini implants can be used in high dense bone as in mandible to increase the stability and success rate of implants. Bone density and implant length play a role in deflection of mini implant from its intended path of insertion. The relationship of the insertion pathway with the adjacent structures has to be evaluated in order to reduce the iatrogenic damage.

## Conclusions

Longer mini implants can be used in less dense bone as in maxilla, whereas shorter mini implants can be used in high dense bone as in mandible to increase the stability and success rate of implants. Bone density and implant length play a role in deflection of mini implant from its intended path of insertion.
